# TPE seems to be a treatment that may improve outcomes by effectively removing fibrin degradation products and restoring coagulation status: fact or fiction?

**DOI:** 10.1186/s13054-020-03309-3

**Published:** 2020-10-06

**Authors:** Patrick M. Honore, Leonel Barreto Gutierrez, Luc Kugener, Sebastien Redant, Rachid Attou, Andrea Gallerani, David De Bels

**Affiliations:** ICU Department, Centre Hospitalier Universitaire Brugmann-Brugmann University Hospital, Place Van Gehuchtenplein, 4, 1020 Brussels, Germany

Gucyetmez et al., noting that elevated D-dimer levels have been found as a predictor for mortality in patients with COVID-19 pneumonia, concluded that therapeutic plasma exchange (TPE) seems to be a treatment that may improve outcomes by effectively removing fibrin degradation products (FDPs) and restoring coagulation status [1]. We are not sure that the authors have demonstrated the point that they intended to make. They propose the use of TPE to remove FDPs, with the rationale that while unfractionated heparin (UFH) and low molecular weight heparin (LMWH) decrease production of FDPs, they cannot contribute to the metabolization of existing FDPs [1]. After propensity score matching, the mortality rate, in the patients with D-dimer level ≥ 2 mg/L, was 8.3% in patients who received TPE (TPE +) versus 58.3% in those who did not (TPE −), with no thromboembolic events detected in either sub-group [1]. While there was a reduction in the D-dimer levels in the TPE + group and not in the TPE − group, this cannot automatically be assumed to be the underlying cause of the decreased mortality rate. The cause of death is important information that has been omitted from this paper. Furthermore, “treating the numbers” does not necessarily equate to an improvement in the status of the patient. It is also important to note that TPE has the potential to cause harm by diluting or attenuating the patient’s adaptive response to infection via depletion of immunoglobulins and complement components 3 and 4 [2]. In the case of patients with COVID-19, TPE will remove the protective antibodies formed by the patient, which is not desirable. Indeed, TPE may not restore immune homeostasis but may rather aggravate immunoparalysis [3]. Finally, given the variety of additional treatments (e.g., antiviral drugs, cytokine filters, steroids) that the patients in the study received, how can one be certain of which treatment(s) ultimately influenced mortality? A randomized controlled trial is needed to truly assess the therapeutic efficacy of TPE in patients with COVID-19 and coagulation activation. All patients should receive standard supportive intensive care without any of the recently proposed treatments for COVID-19, with the exception of dexamethasone. The treatment group would receive three daily sessions of TPE. The prognostic model at admission, the daily severity measure, and outcome measures including detection of thromboembolic events and mortality would be clearly defined by the investigators as in any good quality intensive care trial.

## Data Availability

Not applicable.

## Abbreviations

TPE: Therapeutic plasma exchange
FDPs: Fibrin degradation products
UFH: Unfractionated heparin
LMWH: Low molecular weight heparin
TPE +: Patients who received TPE
TPE −: Patients who did not receive TPE
