# Supplementary material for: TPE seems to be a treatment that may improve outcomes by effectively removing fibrin degradation products and restoring coagulation status: fact or fiction?
Source: Crit Care. 2020 Oct 6;24:599. doi: 10.1186/s13054-020-03309-3 (PMC7538267; doi:10.1186/s13054-020-03309-3)
Supplement: Related texts

# Reply: Authors’ response

**Authors:** Bulent Gucyetmez

Dear Editor,

We would like to thank Honore et al. for their considerable comments. Firstly, we should emphasize three important points about our study: (1) we investigated the effect of therapeutic plasma exchange (TPE) on overall mortality, not mortality predictors, (2) we emphasized that “major thromboembolic events” were not detected, not only thromboembolic events, and (3) we did not mention the cause of deaths because all of them were multi-organ failure (MOF) caused by COVID-19 [1]. Even if “major thromboembolic events” are not detected, patients will be under risk in terms of micro-embolisms as long as their D-dimer levels are high and this could be a reason for MOF.

In the study, 95% and 80% of deaths were in GII and GIIb respectively although all therapies except TPE were similar. Additionally, in propensity score matching (PSM), 14 covariates including “the usage of steroid, interleukin blocker and cytokine filter” were matched [1]. Therefore, we think that the result of PSM is acceptable evidence for the effect of TPE on mortality in patients with high D-dimer level although the number of patients are limited.

On the other hand, references which are used by Honore et al. actually support our results [2, 3]. Namely, Rimmer E et al. mentioned the benefits of TPE before its harmful effects in the introduction section. Moreover, despite the possible rare adverse effects of TPE, they concluded that the mortality was decreased by TPE in adult patients with sepsis [2]. In another reference that 54 patients were included, 41 of them were administered steroid plus immunosuppressive agents. Nevertheless, authors emphasized that “88.9% of procedures were carried out without complications” and they opined that “Lower leucocyte counts could have affected increased susceptibility to infections; however, it is difficult to attribute them exclusively to TPE procedures” [3]. Additionally, fresh frozen plasma (FFP) was administered at 1/4 ratio with albumin during TPE in both studies [2, 3]. Dilution is more expected in this technique. Yet, we only used FFP by calculating estimated plasma volume while performing TPE. Lastly, it should not be forgotten that dexamethasone, which was mentioned as a proposed treatment by Honore et al., is also an immunosuppressive agent [4].

The main treatments of COVID-19 pneumonia are antiviral and anticoagulation therapies. However, we still strongly believe that “TPE should be featured as a part of the treatment especially in COVID-19 pneumonia patients with a high risk of thrombosis”.
